# Type 2 diabetes compromises the value of non-invasively measured augmentation index in predicting the severity of coronary artery disease: a hospital-based observational study

**DOI:** 10.1186/s12872-016-0392-2

**Published:** 2016-11-10

**Authors:** Sijing Wu, Yujie Zhou, Yueping Li, Yuyang Liu, Dongmei Shi, Xiaoli Liu, Wei Liu, Yi Yu, Shuo Jia

**Affiliations:** Beijing Anzhen Hospital, Capital Medical University, Beijing Institute of Heart Lung and Blood Vessel Disease, Beijing, China

**Keywords:** Coronary artery disease, Type 2 diabetes mellitus, Augmentation index, SYNTAX score

## Abstract

**Background:**

Central hemodynamic indices have been demonstrated to correlate with coronary artery disease (CAD). However, in the context of type 2 diabetes mellitus (DM), this correlation has not been fully illustrated. Therefore, this study was employed to investigate the impact of DM on the correlation between aortic augmentation index and the severity of coronary artery disease.

**Methods:**

In this study, we analyzed 197 patients who underwent coronary angiography at Anzhen Hospital from September 2015 to January 2016. Central hemodynamics were non-invasively measured with BPro® device (Health STATS, Singapore). The severity of CAD was defined according to SYNTAX scores. Type 2 diabetes was defined according to ADA guidelines. AIx@75 was defined as AIx normalized to a heart rate of 75 bpm. Receiver operating characteristics (ROC) determined the optimal cut-off value of AIx@75 to predict moderate to severe CAD. Multivariate logistic regression analysis evaluated the correlation between central hemodynamic parameters and CAD severity.

**Results:**

Eighty-four (42.6 %) of the studied subjects were diabetic patients. Our findings were that (1) AIx@75 was significantly correlated with SYNTAX. (ROC analyzed AUC: 0.638, 95 % CI 0.555–0.721, *p* < 0.05). The cut-off value of AIx@75 to predict moderate-to-severe CAD as SYNTAX score more than 22 was 71.45. (2) In non-diabetic patients, correlation analysis revealed that AIx@75, augmentation pressure and peak relative time were significantly correlated with CAD severity (*p* < 0.05). After adjustment, AIx@75 remained as the only independent predictor of moderate-to-severe CAD (odds ratio 1.099, 95 % CI 1.028–1.176, *p* < 0.05). (3) In diabetic patients, the correlation between central hemodynamic parameters and the severity of CAD did not exist.

**Conclusions:**

Aortic augmentation index was significantly related to the severity of CAD and was an independent predictor of severe CAD. However, clinical practitioners should note that its value in DM populations was compromised.

## Background

Central pressures are revealed to be better related to cardiovascular events than are peripheral pressures [1]. Furthermore, central pressures are pathologically more relevant than peripheral pressures with CAD [2]. The advancement of non-invasive techniques to measure central blood pressure has allowed for safer and wider application in more patients. Augmentation pressure (AP), aortic augmentation index (AIx) and pulse wave analysis-derived peak relative time (PRT) are markers of arterial stiffness [3]. Recently, non-invasively measured central hemodynamic parameters have been reported to be related with CAD [4, 5]. However, no reliable cut-off value for such parameters has been determined.

Type 2 diabetes mellitus (DM) is a common comorbidity for CAD. Diabetic patients with CAD demonstrate accelerated progression of coronary atherosclerosis, leading to worse clinical outcome [6]. Arterial stiffness can increase in the presence of overt DM. Central hemodynamics is significantly altered in diabetic patients [3, 7]. Yet whether the coexistence of DM exerts any influence on the relationship between central hemodynamic parameters and CAD remains unclear.

Previous studies have failed to elucidate the effect of comorbidities like type 2 diabetes mellitus on the correlation between central hemodynamic indices and CAD. In view of these considerations, we initiated the present study to investigate the correlation between non-invasively measured central hemodynamic parameters and the severity of CAD. Meanwhile, we explored in detail the influence of type 2 diabetes on the correlation.

## Methods

### Study design and patient recruitment

The cross-sectional study was performed at Anzhen Hospital in Beijing. We consecutively included patients who underwent coronary angiography at our center from September 2015 to January 2016. Exclusion criteria: 1. Patients without complete baseline data; 2. Patients whose central pressure measurement was not readable; 3. Patients who had previous coronary intervention or coronary artery bypass grafting, atrial fibrillation, or hemodynamically significant valvular heart disease were excluded. This study was approved by the Institutional Ethics Committee of Anzhen Hospital and all subjects gave written informed consent.

### Measurement of blood pressure and pulse wave analysis

**Fig. 1:**
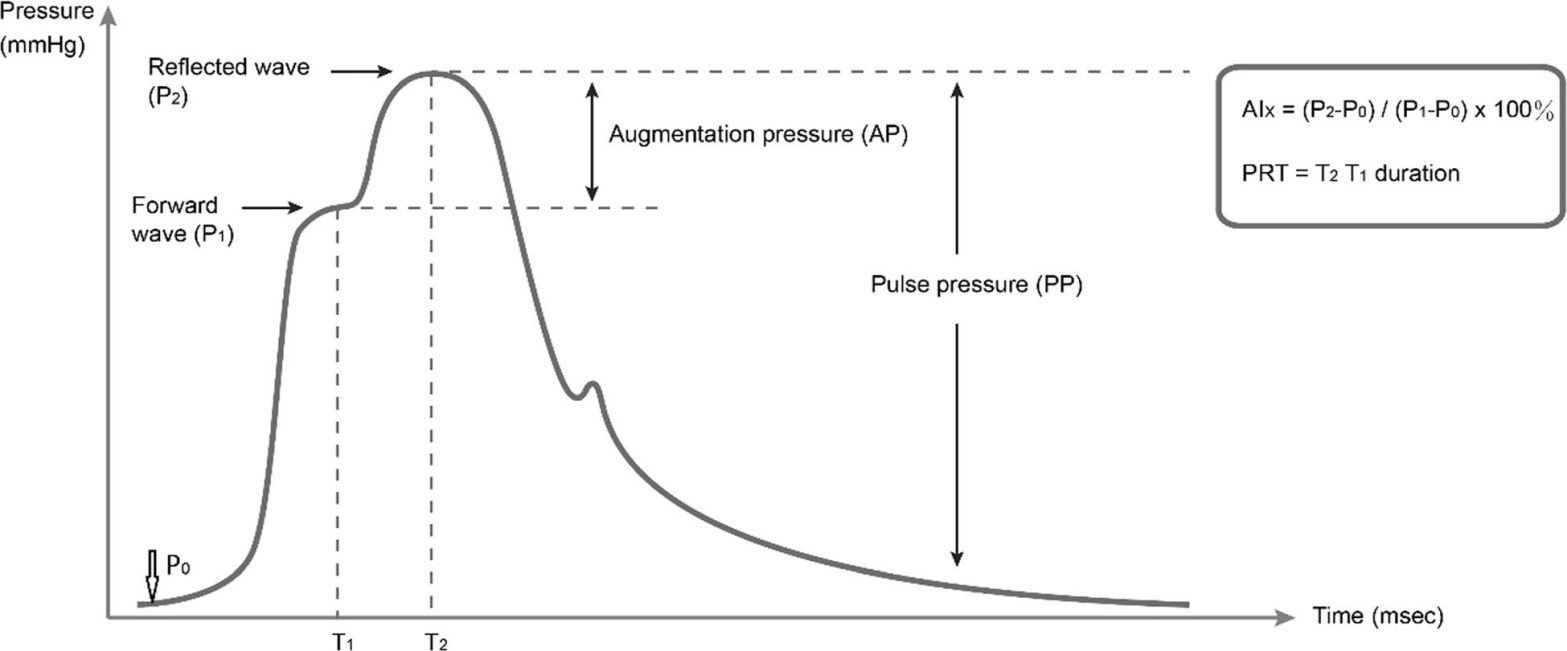
Pressure waveform measurement. AIx = augmentation index PRT = peak relative time

Noninvasive blood pressure and pulse wave analysis were performed prior to coronary angiography with the commercially available BPro® device with A-Pulse CASP software (Health STATS, Singapore). The BPro® device captured the radial waveform, which was subsequently used to generate central systolic pressure by N-point moving average method as described before [8]. Peripheral pressure waveforms were recorded from the wrist using applanation tonometry. After 20 sequential waveforms had been obtained, augmentation pressure (AP), augmentation index (AIx) and peak relative time (PRT) were derived from the measurement of PWA. The central hemodynamic indices are illustrated in Fig. 1. AP was defined as the difference between P1 and P2, AIx was calculated as AP divided by pulse pressure and PRT was described as T2T1 duration. Given that AIx is inversely related to heart rate, we used AIx normalized to a heart rate of 75 bpm (AIx@75) as an alternative. The BPro® device measured arterial pressure waveforms in 10s blocks over the course of 20–30s and the first stable waveform block was used for data analysis. The experienced operator visually inspected the waveforms for anomalies and selected the first stable waveform block, which applied to the criteria of adequate pulse height (100 mV) and pulse length variability (<20 %) [9]. Each patient was measured three times to avoid bias.

### Analysis of coronary angiography

Coronary angiography (CAG) was primarily performed through the trans-radial approach with standardized technique. To reach consensus, all coronary angiograms were visually assessed by at least two experienced interventional cardiologists. The extent and severity of coronary artery disease were determined by SYNTAX scores. The anatomical SYNTAX score was calculated with online calculator version 2.1 at www.syntaxscore.com. The following is 12categorization of CAD severity assessed by SYNTAX score according to guidelines: low as ≤22, moderate as 23–32 and severe as ≥33 [10, 11].

## Statistical analysis

The data were analyzed with the SPSS 15.0 software (Chicago, IL, USA). Continuous variables were presented as mean ± standard deviation (SD) and categorical variables as percentages. Comparisons between groups were made by using the unpaired student *t* test (two-tailed) or Mann-Whitney U-test for continuous data and the Pearson’s χ^2^- or Fisher’s exact test. Spearman’s correlation coefficient (two-tailed) was used for the analysis of the correlation between AIx and SYNTAX scores. The receiver-operating characteristic curve was used to evaluate the predictive accuracy of AIx@75 for moderate to severe CAD; the highest Youden index (J statistic) representing the maximum potential effectiveness was used to determine the optimal cut-off AIx@75 value. Logistic regression analysis was employed for multivariate and univariate analysis. A p value of <0.05 was considered statistically significant.

## Results

### Patient demographics

Our study consecutively enrolled 318 patients. A total of 85, 20 and 16 patients were excluded due to a history of percutaneous coronary intervention, coronary bypass grafting and atrial fibrillation, respectively. The analysis of 197 participants compared 113 non-diabetic with 84 diabetic patients. Baseline characteristics are summarized in Table 1. The mean age of subjects was 59.2 ± 11.1 years old, and 72.1 % were men. The distribution of hypertension, dyslipidemia and current smoking status were not significantly different between groups (*p* > 0.05), nor were laboratory findings and medications.

**Table 1:** Baseline characteristics

	Total (*n* = 197)	Non-diabetic (*n* = 113)	Diabetic (*n* = 84)	*p*-value
Age(years)	59.2 ± 11.1	57.9 ± 11.0	60.9 ± 11.0	0.069
Gender(male), n(%)	142(72.1)	82(72.6)	59(70.2)	0.646
Hypertension, n(%)	128(65)	67(59.3)	61(72.6)	0.063
Hyperlipidemia, n(%)	45(22.8)	21(18.6)	24(28.6)	0.106
Current smokers, n(%)	101(51.3)	59(52.2)	41(48.8)	0.592
BMI(kg/m^2^)	25.2 ± 5.3	25.4 ± 4.9	25.1 ± 5.7	0.684
SYNTAX	16.4 ± 12.2	16.0 ± 11.9	17.0 ± 12.6	0.546
SYNTAX > 22, n(%)	60(30.5)	35(31.3)	25(29.8)	0.823
Lab data				
HbA1c(%)	6.4 ± 1.2	5.7 ± 0.4	7.3 ± 1.3	<0.3*
Triglyceride(mmol/l)	1.8 ± 1.6	1.6 ± 0.8	2.0 ± 1.8	0.073
LDL cholesterol(mmol/l)	2.6 ± 1.0	2.6 ± 1.0	2.5 ± 1.0	0.775
HDL cholesterol(mmol/l)	1.1 ± 0.3	1.1 ± 0.3	1.1 ± 0.3	0.045*
Creatinine(μmol/l)	79.0 ± 17.6	77.8 ± 17.5	80.6 ± 17.7	0.260*
Medications				
ACEI, n(%)	21(10.7)	11(9.7)	10(11.9)	0.641
ARB, n(%)	17(8.6)	6(5.3)	11(13.1)	0.057
β-blockers, n(%)	35(17.8)	21(18.6)	14(16.7)	0.706
Calcium channel blockers, n(%)	44(22.3)	14(12.4)	30(35.7)	<0.3*
Statins, n(%)	32(16.2)	10(8.8)	22(26.2)	0.9*

*p value for comparison between the non-diabetic and diabetic groups; *p* < 0.05 indicates statistical significance. *BMI* body mass index, *LDL* low-density lipoprotein, *HDL* high-density lipoprotein, *ACEI* angiotensin converting enzyme inhibitor, *ARB* angiotensin receptor blocker

### Central hemodynamic indices analysis

Table 2 shows the values of hemodynamic indices. Mean values of central aortic systolic pressure (CASP), central pulse pressure (central PP) and brachial pulse pressure (brachial PP) were significantly higher in the diabetic group (*p* < 0.05).

**Table 2:** Hemodynamic indices

	Total (*n* = 197)	Non-diabetic (*n* = 113)	Diabetic (*n* = 84)	*p*-value
SBP(mmHg)	130.1 ± 17.3	127.8 ± 17.5	133.2 ± 16.6	0.037*
DBP(mmHg)	74.9 ± 11.1	74.8 ± 10.9	75.0 ± 11.5	0.884
CASP(mmHg)	120.3 ± 16.6	118.1 ± 16.5	123.1 ± 16.5	0.044*
MAP(mmHg)	91.9 ± 12.1	91.3 ± 12.8	92.8 ± 11.2	0.387
AP(mmHg)	11.8 ± 7.5	11.9 ± 7.3	11.7 ± 7.9	0.840
Central PP(mmHg)	45.4 ± 13.0	43.5 ± 11.9	48.1 ± 13.9	0.130
Brachial PP(mmHg)	55.2 ± 14.0	53.2 ± 13.2	58.1 ± 14.5	0.130
PR(bpm)	67.7 ± 10.1	67.5 ± 10.3	67.8 ± 9.8	0.750
AIx(%)	77.8 ± 14.5	77.0 ± 14.0	79.0 ± 15.0	0.372
AIx@75(%)	74.9 ± 14.2	74.0 ± 14.3	76.1 ± 13.9	0.317
PRT(ms)	114.5 ± 27.0	116.1 ± 28.1	112.5 ± 25.4	0.374

*p value for comparison between the non-diabetic and diabetic groups; *p* < 0.05 indicates statistical significance. *SBP* systolic blood pressure, *DBP* diastolic blood pressure, *MAP* mean arterial pressure, *PP* pulse pressure, *PR* pulse rate, *CASP* central aortic systolic pressure, *AP* augmentation pressure, *PRT* peak relative time

In the total patient group, univariate analysis revealed that AIx@75 was significantly correlated with SYNTAX score. AIx@75 value to predict the presence of moderate-to-severe CAD was 71.45 (ROC defined AUC 0.638; sensitivity 75 %, specificity 47 %, 95 % CI 0.555–0.721, *p* = 0.4).

**Fig. 2:**
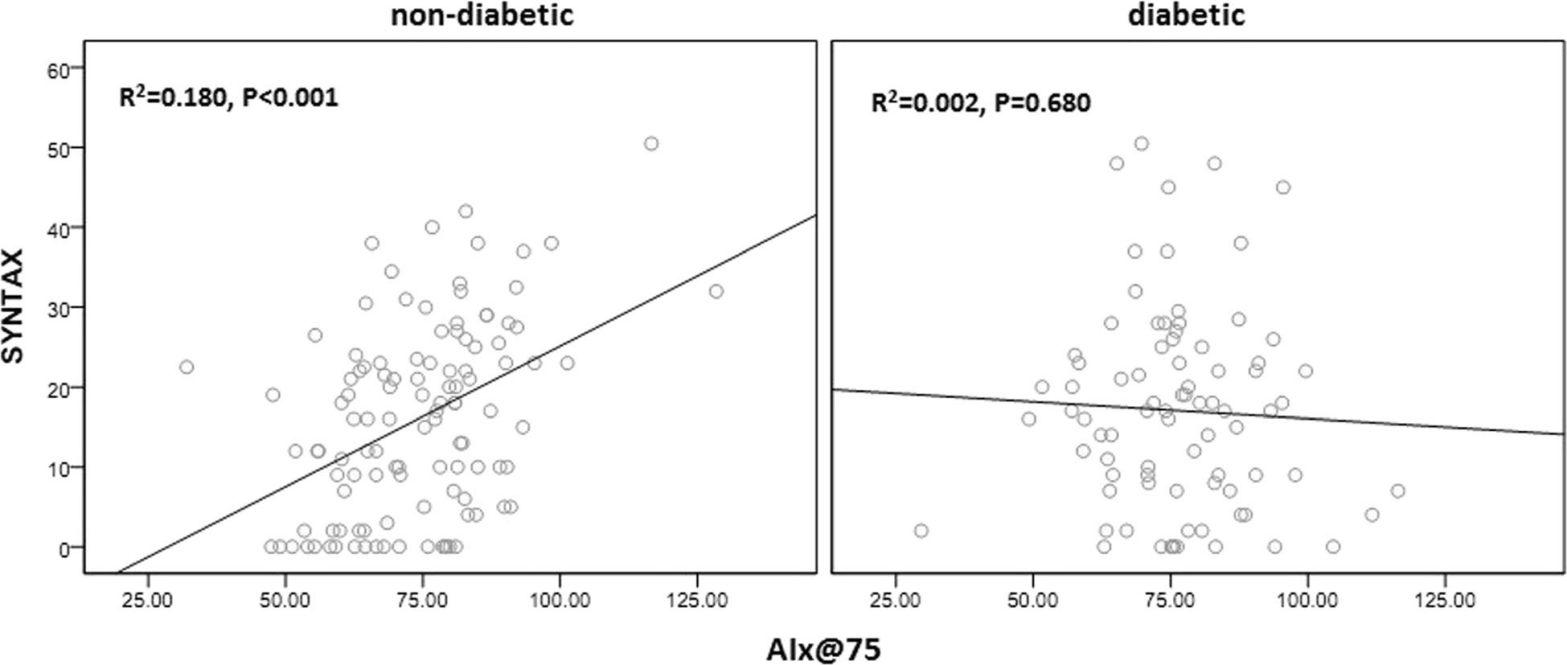
The correlation of AIx@75 and the severity of CAD measured by SYNTAX score according to type 2 diabetes in linear regression analysis

In addition, the correlation between central hemodynamic indices and SYNTAX score differed between groups: univariate logistic analysis showed that higher AIx@75, AP and lower PRT were significantly correlated with SYNTAX score in the non-diabetic group, but not correlated in the diabetic group. Similarly, multivariate logistic analysis showed that higher AIx@75 was significantly correlated with SYNTAX score in the non-diabetic group only (Table 3). Figure 2 demonstrates the linear correlation between AIx@75 and SYNTAX score categorized as non-diabetic (*R* ^2^ = 0.180, *p* < 0.3) and diabetic groups (*R* ^2^ = 0.4, *p* = 0.680).

**Table 3:** Odds ratios for the association between the central hemodynamic parameters and the risk of more severe CAD measured by SYNTAX

Variables	N	SYNTAX > 22, N(%)	Univariate analysis		Multivariate analysis^a^	
			Odds ratio(95 % CI)	*p*	Odds ratio(95 % CI)	*p*
Total	197	60(30.5)				
AIx@75			1.035(1.011–1.059)	0.004		
AP			1.031(0.989–1.075)	0.145		
PRT			0.991(0.980–1.002)	0.120		
Non-Diabetic	113	35(31.3)				
AIx@75			1.064(1.027–1.103)	0.001	1.099(1.028–1.176)	0.006
AP			1.084(1.018–1.154)	0.012	0.938(0.837–1.051)	0.273
PRT			0.985(0.971–1.000)	0.044	1.002(0.984–1.021)	0.800
Diabetic	84	25(29.8)				
AIx@75			1.002(0.969–1.037)	0.885		
AP			0.979(0.921–1.040)	0.491		
PRT			1.001(0.983–1.020)	0.920		

^a^indices that were statistically significant in the univariate logistic regression analysis were included in the multivariate analysis

## Discussion

To the best of our knowledge, this is the first study investigating the impact of type 2 diabetes on the relationship between non-invasively measured central hemodynamic parameters and CAD.

### Augmentation index and coronary artery disease

Cho SW et al. found that AIx was significantly associated with the extent of CAD in the younger group [5]. Tanindi A et al. demonstrated that AIx was correlated with more severe CAD despite patients’ clinical presentation [12]. Similarly, we confirmed that AIx was positively correlated with SYNTAX. Moreover, AIx is an independent predictor of moderate-to-severe CAD. It has been well demonstrated that augmentation index is a parameter reflecting arterial stiffness. However, an established theory explaining why the severity of atherosclerosis in CAD relates to parameters of arterial stiffness is lacking. The concept of bidirectional influence is a possible explanation, namely elevated parameters of arterial stiffness are both a cause and a consequence of atherosclerosis [5, 13]. On the one hand, increased central BP pulsation promotes endothelial damage, leading to the progress of atherosclerosis; On the other hand, diffused atherosclerotic plaques impair the elastic properties of the arterial wall. Physiologically, the reflect wave arrives earlier in the aorta and augments pressure in late systole as aortic stiffness increases. Augmentation of the aortic pressure wave is a manifestation of wave reflection, which can be expressed as the AP [5]. A noteworthy study focused on the time duration analysis and unveiled that augmentation time ratio was related to the presence of CAD [4]. Combined with our finding of the relationship between peak relative time and the severity of CAD, the theory that advanced atherosclerosis significantly increases wave reflection is reasonable.

### The role of diabetes mellitus

We categorized our patients into diabetic versus non-diabetic groups and found that the predictive value of AIx was profound in non-diabetic patients only. Our findings are in line with Agnoletti D et al. [14] who found no specific role of AIx in DM. However, JH Chow et al. [15] recruited both diabetic and non-diabetic patients and revealed that higher AIx@75 was universally associated with PCI among CAD patients. Moreover, MT Schram et al. [16] found that DM was associated with increased AIx. No definitive conclusion has been made regarding the impact of DM on AIx in the CAD population.

AIx is defined as a ratio of augmentation pressure and pulse pressure. Therefore, it is necessary to consider both elements when analyzing its changes. It has been proposed that global stiffening of arteries augments systolic pressure mainly through the increase of wave reflections, whereas local stiffening of proximal aorta amplifies forward pressure wave [4, 17]. Studies concerning arterial pathology in diabetic subjects discovered that DM may lead to preferential increase of large artery stiffness, resulting in lower reflection magnitude [3]. Based on such potential mechanisms and our findings, we suggest that diabetic patients may experience more prominent stiffening of the proximal aorta compared with their non-diabetic peers. As a consequence, diabetes may blunt the correlation between AIx and atherosclerosis-induced arterial stiffness in CAD.

Type 2 diabetes has been proven to be associated with increased central artery stiffness [14, 16, 18]. In a large population-based study, JA Chirinos et al. [19] concluded that DM was associated with increased aortic stiffening, but not with increased carotid stiffness. Subjects with DM demonstrated a decreased reflection magnitude. Prolonged exposure to hyperglycemia can lead to protein glycation, collagen crosslinking and endothelial dysfunction [20]. All of the abnormal changes have deleterious effects on arterial stiffness. Possibly, as DM progresses, these modifications can increase aortic stiffness by a local effect on the arterial wall despite classical determinants of arterial stiffness [14]. Accordingly, AIx may not be the optimal estimation of arterial stiffness and predictor of the severity of CAD in the diabetic population. Future investigations should evaluate the predictive value of AIx in distinct clinical scenarios. As for diabetic CAD patients, experimental research of pathological changes to the aorta is warranted. Previous studies have found that in DM patients, PP was an independent predictor of CAD [19]. Future studies may identify other meaningful predictive indices (such as PP) for diabetic CAD patients. Moreover, central hemodynamic parameters are presumably affected by patient conditions such as impaired renal function [21], so a comprehensive study including all central hemodynamic status-related factors might be valuable in the future.

### Limitations

First, the present study was limited by its relatively small sample size; thus, future research evaluating a larger number of patients is necessary. Second, the study’s observational design, permitted potential confounders to affect the result even after adjustment. Third, long-term follow up studies should be implemented to explore the possible predictive value of AIx for cardiovascular outcomes in non-diabetic patients. Forth, the current study didn’t record patient-reported duration of DM and DM-related organ damage. It will be valuable to investigate the impact of the duration or status of the disease on the correlation in future research.

## Conclusions

Aortic augmentation index is significantly related to the severity of CAD and is an independent predictor of more severe CAD. However, its value in DM population was compromised, which might be related to DM-specific arterial changes and clinical practitioners should be aware of the fact.

## Abbreviations

AIx: Augmentation index
AIx@75: Augmentation index normalized to heart rate of 75 bpm
AP: Augmentation pressure
CAD: Coronary artery disease
CASP: Central aortic systolic pressure
DM: Type 2 diabetes mellitus
PRT: Peak relative time
